# Involvement of CRMP2 Phosphorylation in Amyloid Beta-induced Tau Phosphorylation of Hippocampal Neurons in Alzheimer’s Disease Mouse Model

**DOI:** 10.1007/s12035-025-04721-y

**Published:** 2025-02-01

**Authors:** Daisuke Noguchi, Naoto Watamura, Miyu Nikkuni, Takaomi C. Saido, Yoshio Goshima, Toshio Ohshima

**Affiliations:** 1 Department of Life Science and Medical Bioscience, Laboratory for Molecular Brain Science, Waseda University, 2-2 Wakamatsu-Cho, Shinjuku-Ku, Tokyo 162-8480 Japan; 2 Laboratory for Proteolytic Neuroscience, RIKEN Center for Brain Science, 2-1 Hirosawa, Wako-shi, Saitama 351-0198 Japan; 3 Department of Molecular Pharmacology and Neurobiology, Yokohama City University Graduate School of Medicine, Yokohama, 236-0004 Japan

**Keywords:** Alzheimer’s disease, Tau, Amyloid peptide, Phosphorylation, Microglia

## Abstract

Alzheimer’s disease (AD) is the most common form of dementia, characterized by amyloid-β (Aβ) deposition and the formation of neurofibrillary tangles composed of hyperphosphorylated tau. Collapsin response mediator protein 2 (CRMP2), a microtubule (MT)-binding protein, regulates MT dynamics and is phosphorylated at Ser522 by cyclin-dependent kinase 5. Previous studies have shown increased CRMP2 phosphorylation at Ser522 (CRMP2-pSer522) in early AD stages and AD mouse models, where it colocalizes with phosphorylated tau. However, the role of CRMP-pSer522 in AD pathology remains unclear. In this study, we generated double transgenic mice by crossing tau Tg (PS19) mice and CRMP2 S522A knock-in (CRMP2KI) mice, in which S522 of CRMP2 was replaced with alanine to create a phospho-defective model. No significant change in tau phosphorylation was observed in the hippocampus of tau Tg; CRMP2KI mice compared to tau Tg littermates. However, when Aβ25-35 oligomers were injected into the hippocampus, tau phosphorylation was significantly reduced in Aβ-injected tau Tg; CRMP2KI mice compared to Aβ-injected tau Tg controls. These findings suggest that CRMP2 phosphorylation at Ser522 promotes Aβ-induced tau phosphorylation in this mouse model of AD.

**Supplementary Information:**

The online version contains supplementary material available at 10.1007/s12035-025-04721-y.

## Introduction

Alzheimer’s disease (AD) is a progressive neurodegenerative disorder and the leading cause of dementia in the elderly population worldwide [1]. The hallmarks of AD include the accumulation of extracellular senile plaques composed of amyloid beta (Aβ) and intracellular neurofibrillary tangles (NFTs) formed by hyperphosphorylated tau protein [2]. Aβ is produced by the sequential cleavage of amyloid precursor protein (APP), while tau is a microtubule-associated protein (MAPT) predominantly expressed in neurons of the central nervous system [3]. Under physiological conditions, tau stabilizes microtubules (MTs), but under pathological conditions, it undergoes several post-translational modifications (PTMs), including phosphorylation, truncation, nitration, glycation, glycosylation, ubiquitination, and polyamidation [4]. These aberrant PTMs cause tau to disassociate from MTs and aggregate into insoluble species, such as tau oligomers and filaments, which contribute to AD pathology [4–6].

Tau phosphorylation occurs in distinct stages during disease progression. For instance, phosphorylation at residues T181, T217, T231, or S396 occurs in the early stages of preclinical AD, while phosphorylation at S199, S202/T205, and S422 appears in the later stages (Braak stage V/VI) [7–9]. Aβ accumulation precedes tau pathology, suggesting that Aβ may trigger the elevation of tau PTMs [10, 11]. Although the precise mechanisms remain unclear, Aβ-induced neuroinflammation, mediated by activated microglia [12, 13] and astrocytes [14–16] is thought to exacerbate tau pathology. However, some studies propose that Aβ-independent factors, such as genetic risk and metabolic pathways, can also regulate tau pathology in AD models, including transgenic mice and patient-derived human induced pluripotent stem cell (iPSC) models [17, 18].

Collapsin response mediator protein 2 (CRMP2) is a microtubule-binding protein that regulates MT dynamics, particularly MT elongation [19]. CRMP2 is phosphorylated at Ser522 by Cyclin-dependent kinase 5 (Cdk5) [20]. This phosphorylation serves as a priming event, enabling further phosphorylation by glycogen synthase kinase 3 beta (GSK3-β) at T518, T514, and T509, leading to the dissociation of CRMP2 from MTs [20]. Importantly, phosphorylated CRMP2 has been detected in NFT in both AD patients [21] and transgenic AD mouse models [22]. Interestingly, CRMP2 phosphorylation occurs prior to tau phosphorylation in AD brains and animal models [23], yet its precise pathological role remains unclear.

In this study, we developed a CRMP2 knock-in (CRMP2KI) mouse model to investigate the role of CRMP2 phosphorylation in AD pathology. The CRMP2KI model is a phospho-defective, where Ser522 is substituted with alanine, preventing phosphorylation at this site and the subsequent GSK3-β-mediated phosphorylation at other critical sites [24]. We crossed CRMP2KI with tau transgenic (tau Tg) mice, specifically the PS19 strain, which exhibits tau pathology [25], to generate tau Tg; CRMP2KI mice. Our initial analysis showed no significant difference in AT8-positive tau staining between tau Tg and tau Tg; CRMP2KI mice. However,, the role of CRMP2 phosphorylation in modulating tau phosphorylation remains unclear. To further investigate, we administered Aβ peptide injections to the brain of tau Tg and tau Tg; CRMP2KI mice. The results indicate that Aβ-induced tau phosphorylation was attenuated in tau Tg; CRMP2KI mice compared to tau Tg controls. Taken together, our findings provide the first evidence that inhibition of CRMP2 phosphorylation at Ser522 reduces Aβ-induced tau phosphorylation, suggesting a novel mechanism through which CRMP2 modulates tau pathology in AD.

## Materials and Methods

### Animals

All animal experiments were conducted in compliance with protocols approved by the Institutional Animal Care and Use Committee at Waseda University (Approval Nos. 2021-A022, 2022-A032, and A23-051). Mice were housed under standard laboratory conditions with a 12-h light/dark cycle and had ad libitum access to food and water. Heterozygote P301S tau transgenic mice (PS19 line), originally generated in a mixed-genetic background [25], were backcrossed to the C57BL/6 J strain and maintained on this background.

To generate P301S tau Tg; CRMP2KI/KI mice (referred to as tau Tg; CRMP2KI), male P301S tau Tg mice were crossed with female CRMP2KI/KI mice, producing F1 offspring heterozygous for CRMP2KI (P301S tau Tg; CRMP2KI/ +). These F1 P301S tau Tg; CRMP2KI/ + mice were bred with CRMP2 KI/KI mice to generate P301S tau Tg; CRMP2KI/KI mice. In this mating scheme, the P301S tau Tg was consistently maintained in the hemizygous state to ensure uniform transgene copy number. Mice with the same genetic background, including P301S tau Tg and P301S tau Tg; CRMP2KI/KI, were used for subsequent analyses to minimize genetic variability.

### Amyloid Beta 25–35 Preparation

Aβ25–35 peptide monomers were obtained from Peptide Institute Inc. (Japan). The peptides were dissolved in sterile water at a concentration of 1 µg/µL, aliquoted, and stored at −20℃ until use. To induce oligomerization, the dissolved Aβ peptides were incubated at 37℃ for 4 days, then aliquoted and stored at −80℃ [26]. Sterile phosphate-buffered saline (1xPBS) was prepared and used as a control in the experiments.

### Intracerebral Stereotaxic Injection

Wild-type (WT), CRMP2KI, tau Tg, and tau Tg; CRMP2KI male mice (6–7 months old) were anesthetized using isoflurane (2–3% for induction and 1–2% for maintenance) and placed in a stereotaxic apparatus (Narishige SR-5 M-HT). Under aseptic surgical conditions, a midline incision was made above the designated injection site, and a segment of the cranial bone overlying the target area was carefully removed using a surgical drill.

Aβ25–35 or sterile PBS (control) was unilaterally injected into the dentate gyrus (DG) of the hippocampus. The injection coordinates were as follows: anterior/posterior: −1.94 mm, medial/lateral: + 1.4 mm, dorsal/ventral: −2.2 mm from the brain surface. Two minutes after the needle insertion to the targeted injection site, the solution was infused at a controlled flow at a rate of 1 µL/min at a volume of 3.0 µL per brain. A 5-µL Hamilton syringe fitted with a 10-gauge beveled needle and connected to an injector pump was used for the infusion. The syringe was secured to the stereotactic frame to ensure the stability during the injection process. To enhance the diffusion, the needle was kept in place for an additional 2 min after the infusion was complete. Subsequently, the incision site was sutured, and the mice were allowed to recover from anesthesia on a 37℃ heating pad before being returned to a sanitized housing environment.

At 30 days post-injection, the mice were perfused with 4% paraformaldehyde (PFA) in PBS and their brains were harvested for further analysis.

### Histological Analysis

All mice used for immunohistochemistry were perfused with 4% PFA in PBS following intraperitoneal injection of a triple anesthesia cocktail (medetomidine hydrochloride 0.3 mg/kg, midazolam 4 mg/kg, and butorphanol tartrate 5 mg/kg). The brains were dissected and post-fixed overnight (O/N) in 4% PFA/PBS. Subsequently, the brains were rinsed in 1xPBS at 4 °C O/N, followed by sequential incubation in 10% sucrose/PBS and 20% sucrose/PBS for 4–8 h.

Brain samples were embedded in a mixture of Tissue Tek optimal cutting temperature (OCT) compound (Sakura) and 20% sucrose (2:1 ratio) for cryostat sectioning. Coronal Sects. (15 µm thick) were prepared using a Cryocut 1800 cryostat (Leica), mounted on MAS-coated glass slides (Matsunami), and stored at −20℃.

Tissue sections were washed in PBS for 30 min and blocked in 3% bovine serum albumin (BSA, SIGMA) diluted in 0.1% Triton X-100 (Wako) in PBS (PBSTr) for 10 min. Antigen retrieval was performed to unmask antigen epitopes by heating sections in citrate buffer (1.47 g citrate in 500 mL water) using a microwave or autoclave. Sections were then blocked in 0.01% PBSTr for 1 h at room temperature (RT), followed by incubation with the primary antibody at 4℃ O/N. After three 10-min washes in 0.01% PBSTr, the sections were incubated with secondary antibodies (Alexa Fluor 594 goat polyclonal antibody (pAb) anti-mouse IgG, Abcam, and Alexa Fluor 647 donkey pAb anti-rabbit IgG, Abcam) for 1 h at RT. The slides were then washed three times with 0.01% PBSTr, dried, and mounted using Fluormount™ Aqueous Mounting Medium (Sigma-Aldrich).

Fluorescent images were acquired using an inverted confocal laser microscope (FV3000, Olympus) equipped with UPlanSApo 40 × oil immersion objective lens (NA 0.95). Images were acquired as z-stacks with eight slices at 1.4-µm intervals. Immunostained signals were analyzed using NIH ImageJ software. The mean gray value of immunolabeling was measured by subtracting the background signal.

### Antibodies

Mouse monoclonal anti-phospho-tau (S202, T205) (AT8; RRID: AB_223647) and anti-total tau (Tau-5; RRID: AB_2536235) antibodies were purchased from Invitrogen. Rabbit polyclonal anti-Iba1 antibody (RRID: AB_839504) was purchased from FUJIFILM. Rabbit polyclonal anti-CRMP2 phospho-T509 antibody (Rabbit/IgG1, 1:500) recognizes the pT509 epitope of CRMP2 [24].

### Statistical Analysis

Statistical analyses were conducted using GraphPad Prism 6 software. An unpaired two-tailed Student’s *t*-tests with Welch’s correction was applied for pairwise comparisons, while one-way analysis of variance (ANOVA) with Tukey’s post-hoc test was used for multiple comparisons. Data are presented as the mean ± standard error of the mean (SEM).

## Results

### No Significant Alteration of Tau Phosphorylation in Hippocampal Neurons in Tau Transgenic Mice Phospho-Defective at Ser522

To investigate the role of CRMP2 phosphorylation in tau pathology, we generated a novel transgenic mouse model, tau Tg; CRMP2KI, by crossing P301S tau Tg mice (PS19 line) [25] with CRMP2KI mice [24]. Previous studies have demonstrated that tau pathology in P301S tau Tg mice develop in an age-dependent manner with tau hyperphosphorylation contributing to neuronal loss and brain atrophy at 9–12 months of age [25]. Based on this, we analyzed the hippocampus of 12-month-old male mice using the anti-phospho-tau (S202, T205) AT8 antibody to assess tau phosphorylation.

**Fig. 1:**
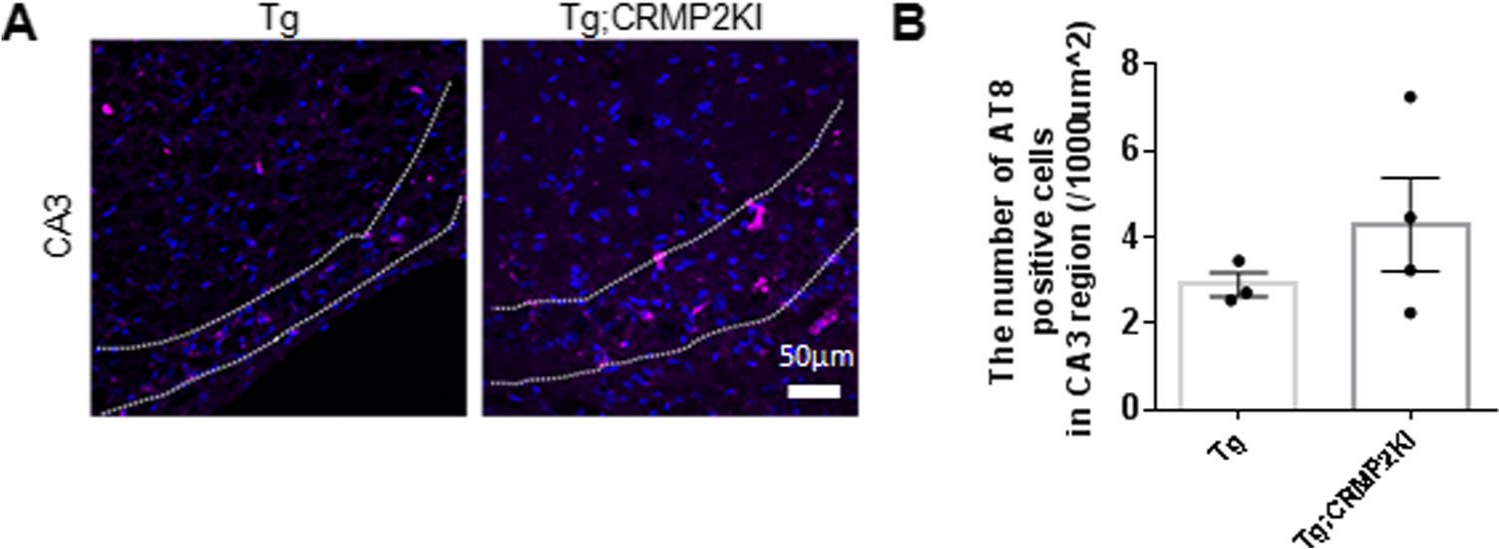
No significant alteration in the number of AT8-positive cells in the CA3 between tau Tg and tau Tg; CRMP2KI mice. **A** Representative immunostaining of pathological tau using AT8 antibody (magenta) and Hoechst nuclear counterstaining (blue) in the CA3 regions of the hippocampus in 12-month-old tau Tg, and tau Tg; CRMP2KI mice. The dotted white lines indicate pyramidal cell layer of CA3. Scale bar 50 µm. **B** Quantification of AT8-positive cells in as a percentage of the area occupied in the hippocampal CA3 region from tau Tg, and tau Tg; CRMP2KI mice showing no significant alteration. Data expressed as mean ± standard error of the mean (SEM). A two-tailed t-test was performed. Tau Tg, *n* = 3: tau Tg; CRMP2KI, *n* = 4)

The number of AT8-positive cells was quantified in the CA3 pyramidal cell layer of the hippocampus (Fig. 1A). Significant accumulation of AT8-positive cells was observed in both tau Tg and tau Tg; CRMP2KI mice. However, no statistically significant difference in the density of AT8-positive cells was detected between tau Tg and tau Tg; CRMP2KI mice at 12 months of age. These findings raised the possibility that the primary tau pathology model may lack sufficient sensitivity to detect the impact of CRMP2 phosphorylation at Ser522 in AD pathogenesis. This suggest that a model incorporating both amyloid and tau pathologies may provide a more appropriate framework to assess the effects of CRMP2 S522A phospho-defective mutation.

### CRMP2 Phosphorylation at T509 is Upregulated Following Aβ Peptide Injection

Next, we injected mice with Aβ peptides to analyze Aβ-induced tau pathology in vivo. Our previous study has demonstrated that intracerebroventricular injection of Aβ25–35 peptide significantly increased CRMP2 phosphorylation at Ser522 in the CA1 region of WT mice [26] and in 18-month-old Tg2576 mice exhibiting amyloid pathology [27, 28]. To investigate the impact of Aβ injection on the CRMP2 phosphorylation, we used an anti-phospho-CRMP2 (T509) antibody that detects CRMP2 phosphorylation at the GSK3-β site (T509). The immunohistochemistry result of the mouse brains harvested 30 days after Aß25–35 injection suggested a significant increase in phosphorylated CRMP2 (T509) intensity in the ipsilateral DG granule cells and CA3 pyramidal neurons of Aβ25–35-injected mice compared with PBS-injected controls (Supplemental Fig. 1). While a non-significant increase in phosphorylated CRMP2 (T509) was observed in the ipsilateral CA1 pyramidal cells, the overall findings indicate that Aβ injection induced a general increase of phosphorylated CRMP2 (T509) in the hippocampus of WT mouse.

### Genetic Inhibition of CRMP2 Phosphorylation Regulates Tau Phosphorylation Induced by Aβ25–35 Injection

We hypothesized that CRMP2 phosphorylation at S522 could be a critical event linking Aβ and tau pathologies. To test this hypothesis, we injected Aβ25–35 peptide or PBS into the DG of 6–7-month-old WT, CRMP2KI, tau Tg, and tau Tg; CRMP2KI mice. At 30 days post-injection, we performed immunohistochemical analysis using the phospho-tau AT8 antibody (pS202/T205) to evaluate the extent of tau phosphorylation across different hippocampal regions.

**Fig. 2:**
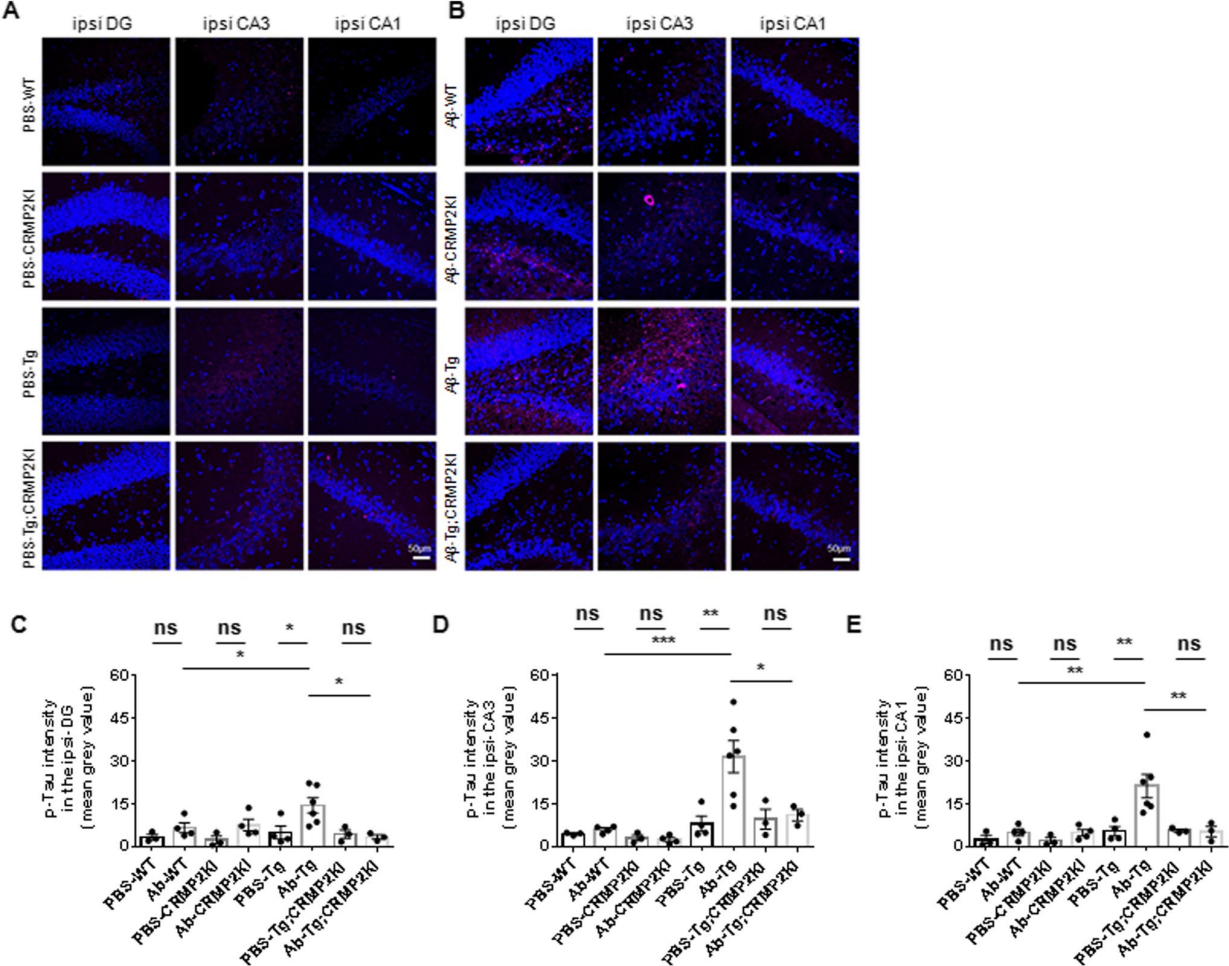
Aß-induced tau phosphorylation is alleviated in the hippocampus of tau Tg; CRMP2KI. (A-B) Representative images of immunostaining with anti-AT8 antibody (magenta) in the ipsilateral hippocampus from PBS- (**A**) or Aβ-injected (**B**) WT, CRMP2KI, tau Tg (Tg), and tau Tg; CRMP2KI (Tg; CRMP2KI) mice. Scale bar = 50 µm. (C-E) Quantification of the mean gray value of AT8-positive intensity in the ipsilateral DG (**C**), CA3 region (**D**) and CA1 region (**E**). There was a remarkable increase in AT8-positive tau in Aß-injected tau Tg mice. The other cohorts showed no significant difference between PBS- and Aβ-injections. Tukey’s multiple comparison test with Geisser-Greenhouse correction used to compare multiple groups. Data expressed as mean ± standard error of the mean (SEM). PBS-WT, *n* = 3; Aß-WT, *n* = 4; PBS-CRMP2KI, *n* = 3; Aß-CRMP2KI, *n* = 4; PBS-Tg, *n* = 4; Aß-Tg, *n* = 6; PBS-Tg; CRMP2KI, *n* = 3 and Aß-Tg; CRMP2KI, *n* = 3. ns, not significant. *P <0.05, **P<0.01, and ***P<0.005

Immunofluorescent images acquired using an inverted confocal microscope were analyzed to measure the mean gray value of AT8-positive signals in each hippocampal region (Fig. 2 A, Supplemental Fig. 2 A). Both WT and tau Tg mice displayed some degree of tau phosphorylation in all hippocampal regions 30 days after Aβ25–35 injection. However, tau Tg mice exhibited a significantly higher accumulation of phosphorylated tau compared to Aβ-injected WT mice (Fig. 2 B-D, Supplemental Fig. 2 B-D). Simultaneous analysis of PBS-injected control mice revealed that Aβ25–35 injection led to a markedly higher level of tau phosphorylation in the hippocampus of tau Tg mice compared to PBS-injected tau Tg mice. These findings indicate that Aβ25–35 injection exacerbates tau phosphorylation in tau Tg mice, suggesting a synergistic effect between Aβ and tau pathologies.

### CRMP2 Phospho-Defective Mutation Does Not Alter the Total Tau Levels

To investigate whether CRMP2 phosphorylation at S522 influences Aβ-induced tau spreading, we assessed the total tau levels in the hippocampus of Aβ25–35-injected mice. The intensity of tau-5-positive signals was measured in various hippocampal regions (Supplemental Fig. 3). No statistically significant differences were observed in the intensity of tau-5-positive signals between Aβ-injected mice and PBS-injected mice across all four genotypes (WT, CRMP2KI, tau Tg, and tau Tg; CRMP2KI) (Supplemental Fig. 3). Additionally, no significant differences in total tau levels were detected between Aβ-injected tau Tg mice and Aβ-injected tau Tg; CRMP2KI mice. These findings indicate that Aβ25–35 injection does not alter total tau levels in the hippocampus, suggesting that the phosphorylation of CRMP2 at S522 does not directly affect the accumulation of total tau in this context.

### Alleviated Microgliosis in Aβ-Injected Tau Tg; CRMP2KI Mice Compared to Aβ-Injected Tau Tg Mice

**Fig. 3:**
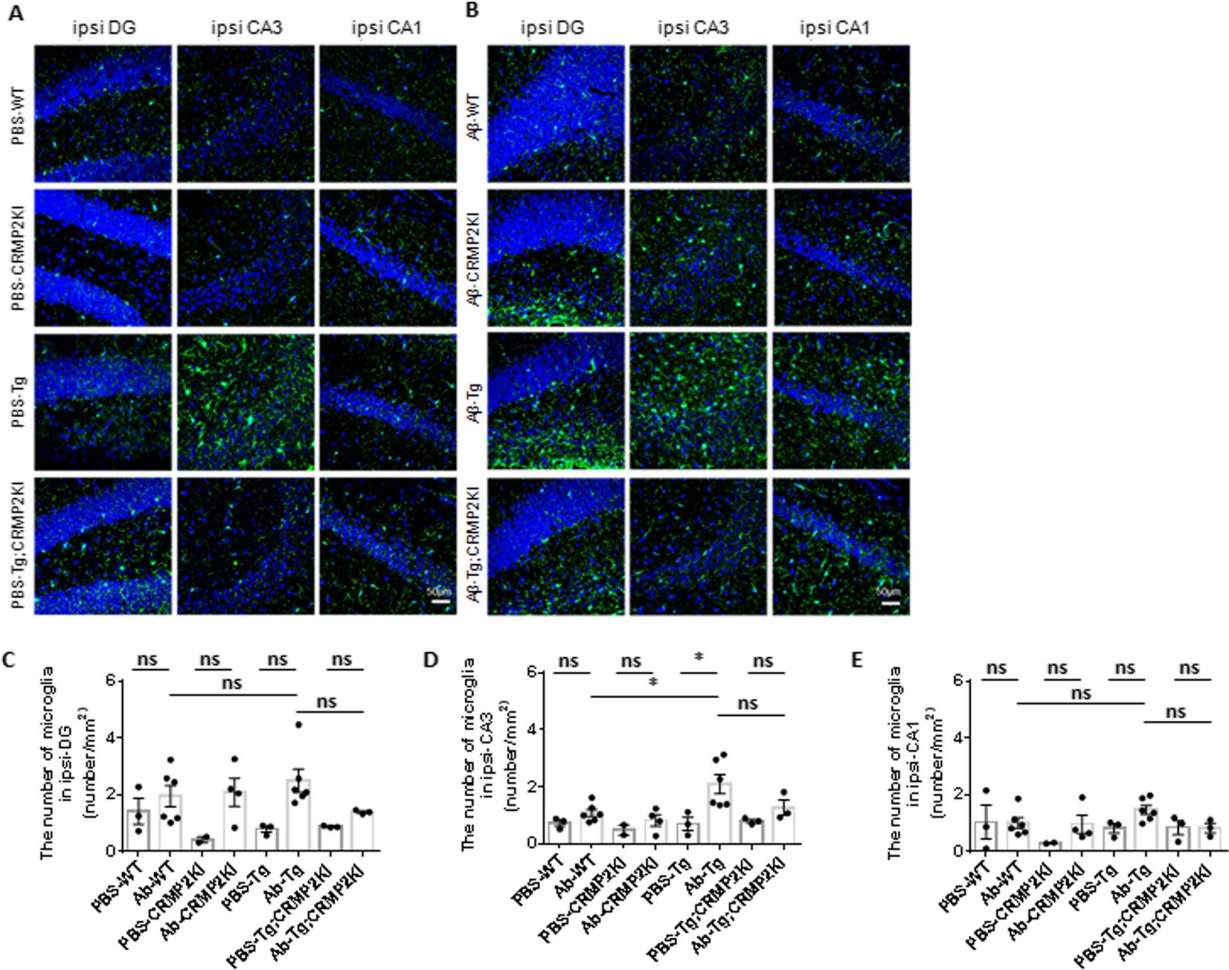
Microglial activation was increased in the hippocampus of Aβ-injected tau Tg mice. (A-B) Representative images of immunostaining with anti-Iba1 antibody (green) in the ipsilateral hippocampus from PBS (**A**) or Aβ-injected (**B**) WT, CRMP2KI, tau Tg (Tg), and tau Tg; CRMP2KI (Tg; CRMP2KI) mice. Hoechst nuclear counterstaining is shown in blue. Scale bar = 50 µm. (C-E) Quantification of the mean gray value of Iba1-positive intensity in the ipsilateral DG (**C**), CA3 region (**D**), CA1 region (**E**). Tukey’s multiple comparison test with Geisser-Greenhouse correction used to compare multiple groups. Data expressed as mean ± standard error of the mean (SEM). PBS-WT, *n* = 3; Aß-WT, *n* = 6; PBS-CRMP2KI, *n* = 2; Aß-CRMP2KI, *n* = 4; PBS-Tg, *n* = 3; Aß-Tg, *n* = 6; PBS-Tg; CRMP2KI, *n* = 3 and Aß-Tg; CRMP2KI, *n* = 3. ns., not significant. *P<0.05

To explore the role of CRMP2 phospho-defective mutation at S522 in mitigating Aβ-induced tau pathology, we examined microgliosis as a potential mechanism interconnecting Aβ and tau pathologies [12, 13]. Immunostaining with an anti-Iba1 antibody was performed to quantify the number of activated microglia in various hippocampal regions (Fig. 3 A). The results showed a globally higher number of microglia in Aβ-injected tau Tg mice compared to Aβ-injected tau Tg; CRMP2KI mice, with a particularly notable increase in the contralateral DG (Fig. 3 B-D). Interestingly, the morphology of microglia differed between the two genotypes. Microglia in Aβ-injected tau Tg; CRMP2KI mice exhibited amoeboid form, except in the ipsilateral DG (Fig. 3 A). Conversely, microglia in Aβ-injected tau Tg mice displayed a relatively ramified morphology (characterized by long branches and small cellular bodies [27]) or a phagocytic form (with thickened and retracted branches [27]), particularly in the contralateral hippocampus (Supplemental Fig. 4). In summary, these findings demonstrate a significant reduction in microglial activation in Aβ-injected tau Tg; CRMP2KI mice compared to Aβ-injected tau Tg mice, suggesting that the CRMP2 phospho-defective mutation at S522 alleviates microgliosis in this pathological context.

## Discussion

Phosphorylated CRMP2 has been reported as a component of NFT in the brains of patients with AD [21] and in AD mouse models [22]. Interestingly, prior biochemical analyses have reported that CRMP2 phosphorylation appears earlier than tau phosphorylation in AD mouse models [23]. These findings suggest a potential role for CRMP2 phosphorylation in the development of tau pathology in AD. However, the relationship between CRMP2 phosphorylation at S522 and tau phosphorylation has remained unclear. In this study, we compared tau pathology in tau Tg and tau Tg; CRMP2KI mice. Our results showed no significant differences in the number of AT8-positive cells in the hippocampus of tau Tg; CRMP2KI compared to tau Tg at 12 months. However, when Aβ25–35 peptide was injected into the DG of the hippocampus, tau phosphorylation was more pronounced in Aβ-injected tau Tg; CRMP2KI mice compared to Aβ-injected tau Tg in the ipsilateral hippocampus. These findings suggest that CRMP2 phosphorylation facilitates Aβ-induced tau phosphorylation.

Previous studies have demonstrated that aggregated Aβ, including oligomeric Aβ, enhances tau phosphorylation both in vitro [29] and in vivo [30, 31]. Consistent with these reports, we observed that Aβ-injected tau Tg mice had a significant increase in AT8-positive labeling compared to PBS-injected tau Tg mice in the hippocampus. Furthermore, hippocampal Aβ-injection in tau Tg mice led to a 2–threefold higher increase of CRMP2 phosphorylation at T509 compared to PBS-injected control mice. Notably, we found a reduction in AT8-positive signals in the ipsilateral hippocampus of Aβ-injected tau Tg; CRMP2KI mice compared to Aβ-injected tau Tg mice. These results indicate that Aß-induced tau phosphorylation is attenuated when CRMP2 phosphorylation at S522 is disrupted, supporting a critical role for CRMP2 in Aβ-mediated tau pathology.

We acknowledge that there are several limitations in this study. First, due to a limited experimental timeline, the impact of CRMP2 S522A on tau seeding remains unclear. Further studies are necessary to compare tau pathology between tau Tg and tau Tg; CRMP2KI mice using tau aggregate inoculation [32] or AAV-tau infection [33] at varying incubation periods. Second, behavioral analyses could provide additional insights into the phenotype of the Aß-injected tau Tg; CRMP2KI mouse model. Despite these limitations, this model is valuable for investigating the role of phospho-defective CRMP2 in Aß-induced tau pathology in vivo.

Microglial activation has been proposed as a plausible mechanism linking Aβ-dependent tau pathology to CRMP2 phosphorylation at S522. Previous studies have demonstrated a direct interaction between microglial activation and Aβ-dependent tau pathology [12, 13]. However, it remains unclear whether phosphorylated CRMP2 at S522 directly correlates with microglial activation. In our study, we observed a trend toward reduced microglial activation in the hippocampus of Aβ-injected tau Tg; CRMP2KI mice compared to Aβ-injected tau Tg mice. These findings are consistent with previous studies that reported reduced microglial activation associated with CRMP2KI S522A in an MPTP-induced PD mouse model [34] and a spinal cord injury model [35]. Since CRMP2 is ubiquitously expressed including microglia and extracellular CRMP2 is known to play important roles in receptor activation [36, 37], further studies are required to clarify the role of microglial activation in the association between CRMP2 phosphorylation and tau pathology.

In conclusion, the novel mouse model of AD featuring both Aß and tau pathologies described here provides a powerful tool for investigating the role of CRMP2 phosphorylation at S522 in Aß-induced tau pathology and its contribution to AD progression.

## Supplementary Information

Below is the link to the electronic supplementary material.

Supplementary file1 (PDF 759 KB)

## Data Availability

The data that support the findings of this study are available from the corresponding author upon request.

## Abbreviations

Aβ: Amyloid beta
AD: Alzheimer’s disease
Cdk5: Cyclin-dependent kinase 5
CRMP2: Collapsin response mediator protein 2
DG: Dentate gyrus
EVs: Extracellular vesicles
huTau: Human Tau
MAP: Microtubule-associated proteins
MTs: Microtubules
NFTs: Neurofibrillary tangles
OCT: Optimal cutting temperature
PTMs: Posttranslational modifications
Tg: Transgenic mice
CRMP2KI: CRMP2 S522A Knock-in/Knock-in mice
