# Supplementary material for: Involvement of CRMP2 Phosphorylation in Amyloid Beta-induced Tau Phosphorylation of Hippocampal Neurons in Alzheimer’s Disease Mouse Model
Source: Mol Neurobiol. 2025 Feb 1;62(6):7413–20. doi: 10.1007/s12035-025-04721-y (PMC12078411; doi:10.1007/s12035-025-04721-y)
Supplement: Supplementary file 1 — Supplementary file1 (PDF 759 KB) [file 12035_2025_4721_MOESM1_ESM.pdf]

Supplemental Information

Involvement of CRMP2 phosphorylation in amyloid beta-induced tau phosphorylation of hippocampal neurons in Alzheimer's disease mouse models

Daisuke Noguchi[1], Naoto Watamura[1], Miyu Nikkuni[1], Takaomi C Saido[2], Yoshio Goshima[3], Toshio Ohshima[1,4]

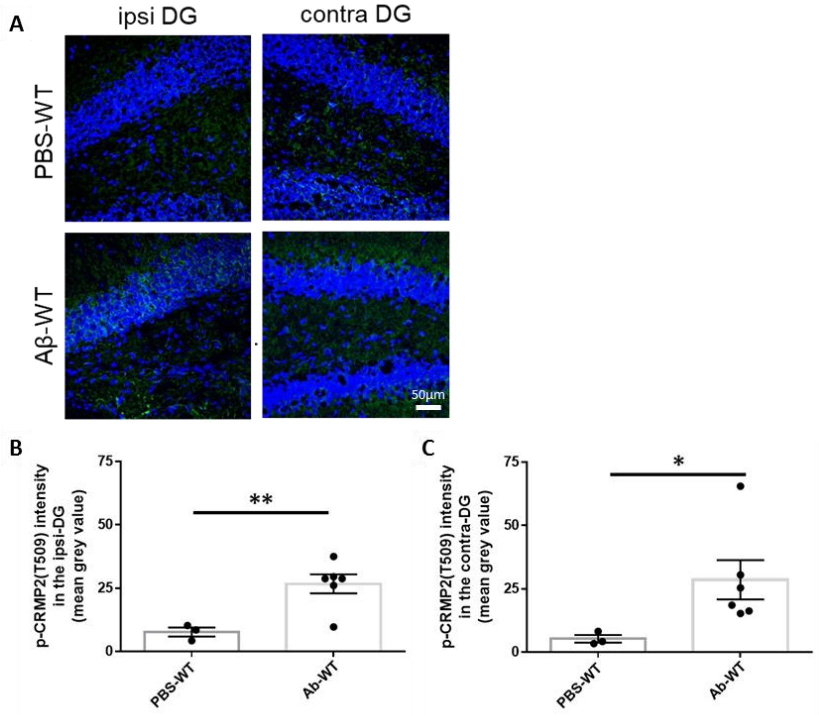

Supplementary Fig1. CRMP2 phosphorylation at T509 was significantly upregulated in the hippocampus of Aß-injected WT mice.

(A) Representative images of immunostaining with anti-CRMP2 (phospho T509) antibody (green) in the ipsilateral and contralateral sides of the hippocampus dentate gyrus (DG) from PBS-injected and Aß25-35-injected WT mice. Nuclear counterstaining is shown in blue. Scale bar 50 µm. (B)-(C) Quantification of the mean gray value of p-CRMP2(T509)-positive intensity in the ipsilateral DG (B) and contralateral DG (C). Groups compared using unpaired two-tailed Student's t-tests with Welch's correction and confidence intervals of 95% used. Data expressed as mean ± standard error of the mean (SEM). PBS-WT, n=3; Aß-WT, n=6. *$P<0.05$, and **$P<0.01$.

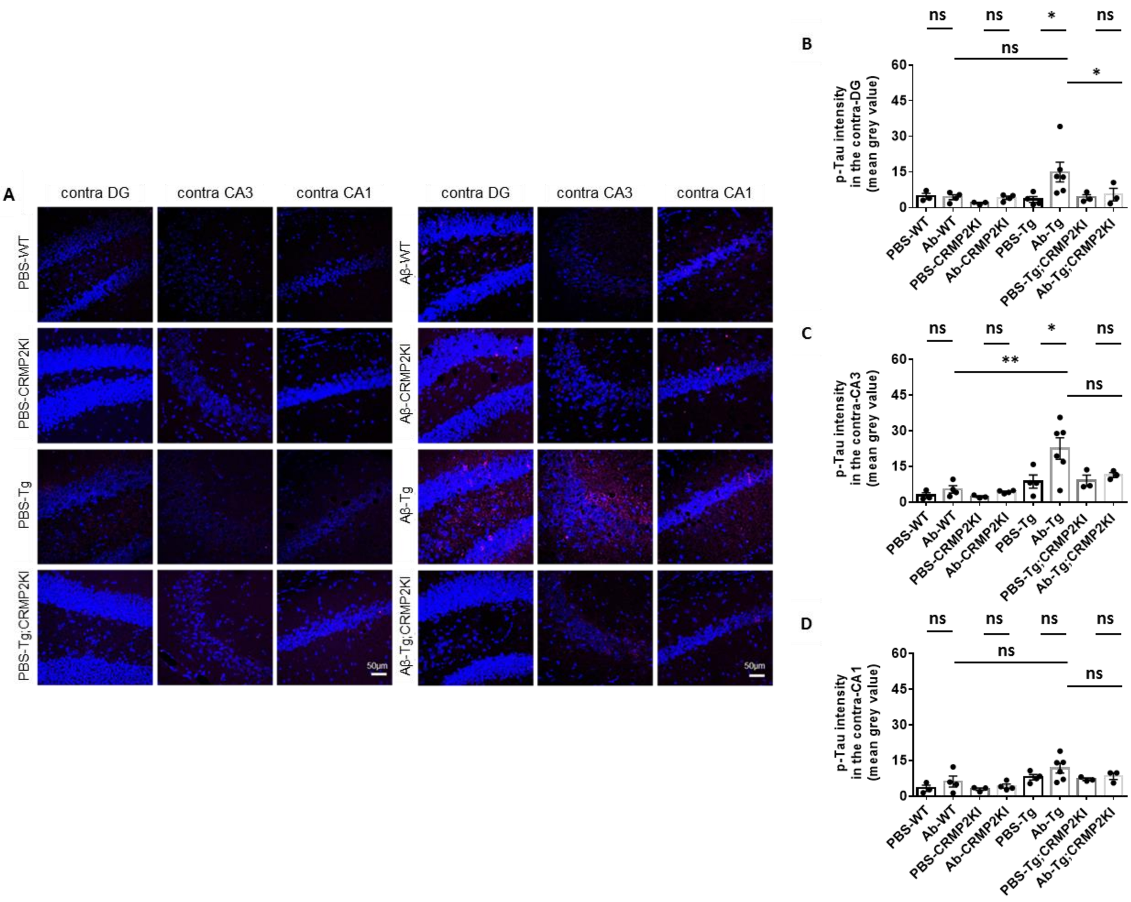

Supplemental Fig2. Tau phosphorylation in the contralateral hippocampus was significantly upregulated Aß-injected tau Tg mice

(A) Representative images of immunostaining with anti-AT8 antibody (magenta) in the contralateral hippocampus from WT, CRMP2KI, tau Tg (Tg), and tau Tg; CRMP2KI mice injected with PBS or Aß$_{25-35}$. Nuclear counterstaining is shown in blue. Scale bar = 50 μm. (B)-(D) Quantification of the mean gray value of AT8 positive intensity in the contralateral DG (B), CA3 region (C), CA1 region (D). Tukey's multiple comparison test with Geisser-Greenhouse correction used to compare multiple groups. Data expressed as mean ± standard error of the mean (SEM). PBS-WT, n=3; Aß-WT, n=4; PBS-2KI, n=3; Aß-2KI, n=4; PBS-Tg, n=4; **Aß-Tg**, n=6; PBS-Tg;2KI, n=3 and Aß-Tg;2KI, n=3. ns, not significant. *P<0.05, **P<0.01.

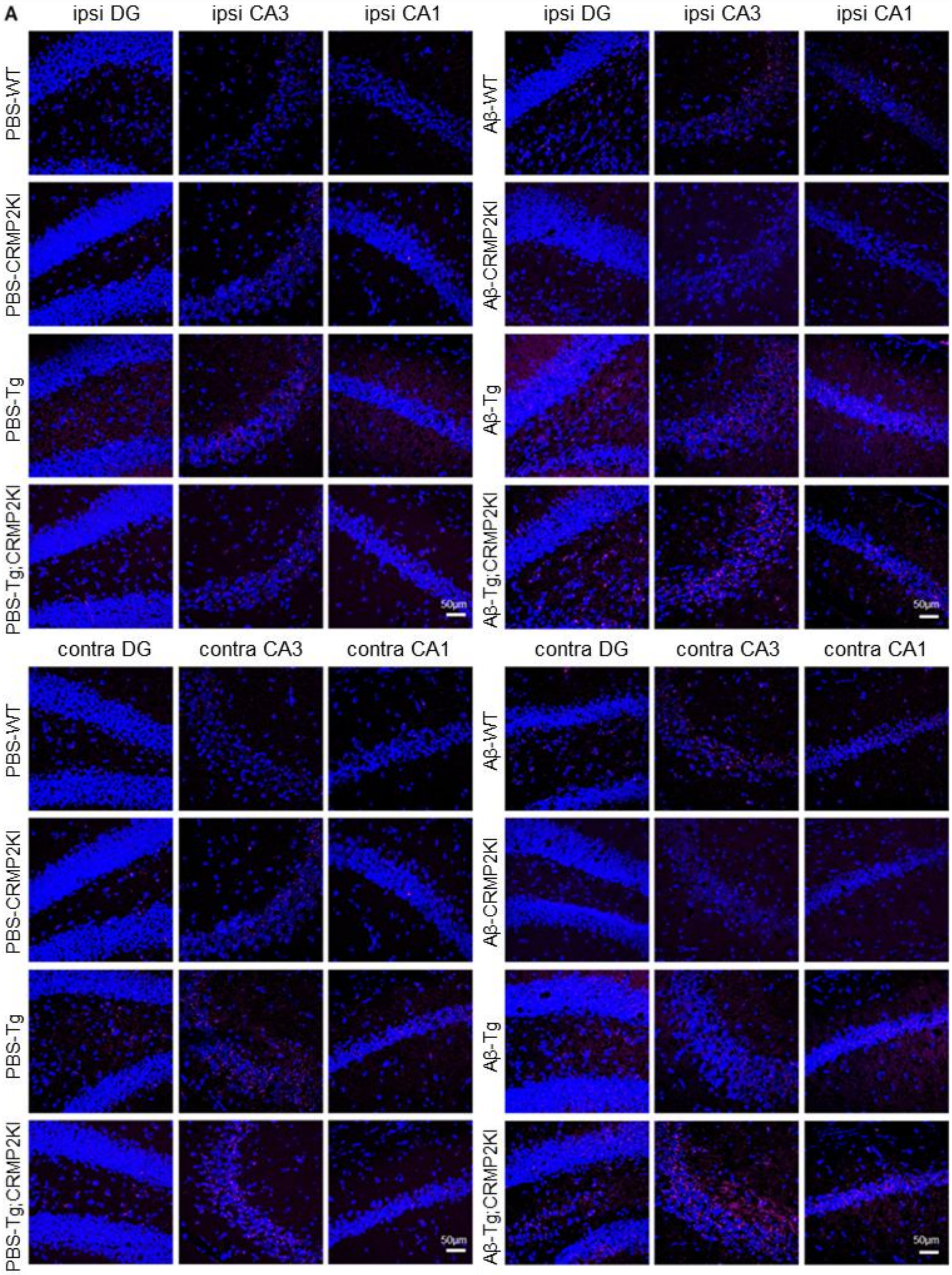
A
ipsi DG
ipsi CA3
ipsi CA1
PBS-WT
PBS-CRMP2KI
PBS-Tg
PBS-Tg;CRMP2KI
Aβ-WT
Aβ-CRMP2KI
Aβ-Tg
Aβ-Tg;CRMP2KI
contra DG
contra CA3
contra CA1
50μm

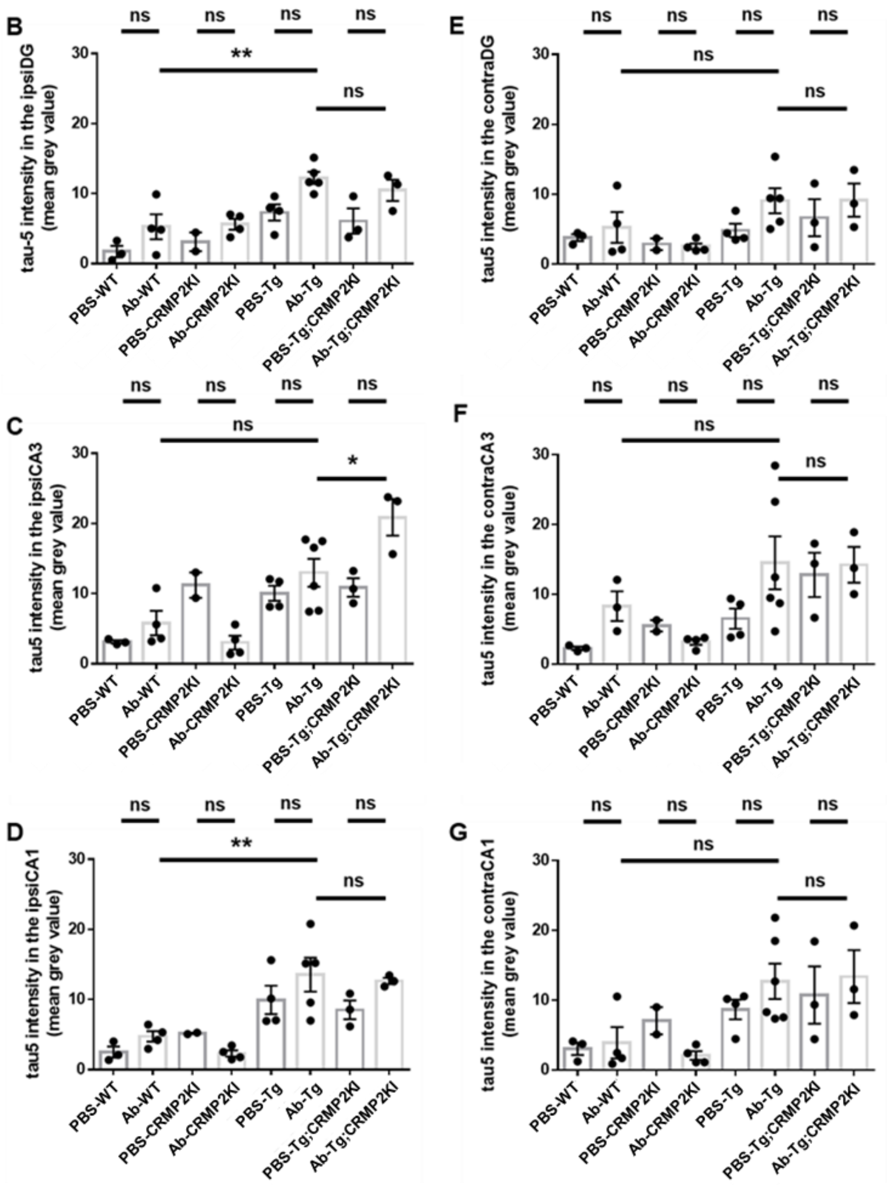

Supplementary Fig3. Immunostaining with anti-tau5 antibody in the hippocampus from PBS and Aß25-35 injection samples.

(A) Images of immunostaining with anti-tau-5 antibody (magenta) in the contralateral hippocampus from WT, CRMP2KI, tau Tg (Tg), and tau Tg; CRMP2KI mice injected with PBS or Aß$_{25\text{-}35}$. Scale bar = 50 µm. (B)–(F) Quantification of the mean gray value of tau-5 positive intensity in the ipsilateral and contralateral DG, CA3, and CA1 region. Tukey's multiple comparison test with Geisser-Greenhouse correction used to compare multiple groups. Data expressed as mean ± standard error of the mean (SEM). PBS-WT, n=3; Aß-WT, n=4; PBS-2KI, n=3; Aß-2KI, n=4; PBS-Tg, n=4; A**ß-Tg**, n=6; PBS-Tg;2KI, n=3 and Aß-Tg;2KI, n=3. ns, not significant. *P<0.05, and **P<0.01.

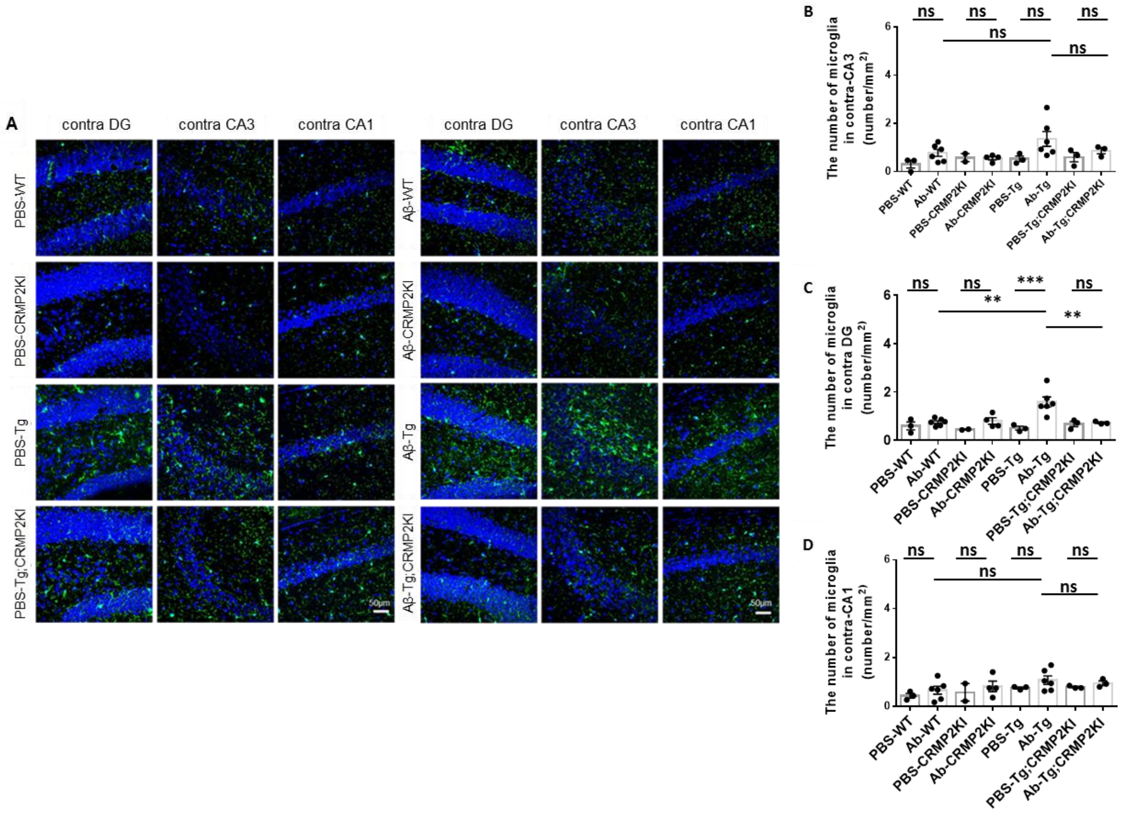

Supplemental Fig4. Microglial activation was alleviated in the contralateral hippocampus DG in Aβ-injected tau Tg; CRMP2KI than in **Aβ-injected tau Tg mice.**

(A) Representative images of immunostaining with anti-Iba1 antibody (green) in the contralateral hippocampus from WT, CRMP2KI, tau Tg (Tg), and tau Tg; CRMP2KI mice injected with PBS or $A\beta_{25\text{-}35}$. Scale bar = 50 μm. (B-D) Quantification of the mean gray value of Iba1-positive intensity in the contralateral DG (B), CA3 (C) and CA1 region (D). Tukey's multiple comparison test with Geisser-Greenhouse correction was used to compare multiple groups. Data are shown as mean ± standard error of the mean (SEM). ns, not significant. **P<0.01, and ***P<0.005.
